# LASER assisted soft tissue procedures for orthodontic treatment

**DOI:** 10.6026/973206300200634

**Published:** 2024-06-30

**Authors:** Bhumika Sehdev, Nityanand Shetty, Ankur Kaul, Prachi Gholap, Shashank S. Gaikwad, Pallavi Mishra, Miral Mehta, Mahesh Ghadage

**Affiliations:** 1 Department of Periodontology, RKDF Dental College and Research Center, Bhopal, M.P., India; 2 Department of Orthodontics, Bharati Vidyapeeth (Deemed to be University) Dental College and Hospital, Navi Mumbai, India; 3 Clinical Director, Auburn Dental Group, Auburn, MA, USA; 4 MDS Prosthodontics & Crown and Bridge, Bharati Vidyapeeth (Deemed to be University) Dental College and Hospital Navi Mumbai, Maharashtra, 400614, India; 5 Department of Oral and Maxillofacial Pathology, Kalinga Institute of Dental Sciences, KIIT Deemed to be University, Patia, Bhubaneswar - 751024, India; 6 Department of Pedodontics, Karnavati School of Dentistry, Karnavati University, Gandhinagar, Gujarat, India; 7 Department of Prosthodontics and Crown and Bridge, Bharati Vidyapeeth (Deemed to be University) Dental College and Hospital, Navi Mumbai, Maharashtra, India

**Keywords:** LASER, orthodontics, soft tissue surgery

## Abstract

Light Amplified Stimulated Emission of Radiation (LASER) therapy has been the subject of numerous researches as an auxiliary method in orthodontic practice. Therefore, it is of interest to assess the clinical evaluation of laser assisted soft tissue procedures for orthodontic treatment. The soft tissue surgical procedures carried out were aestheticre-contouring, gingivectomy, maxillary frenectomy, operculectomy and surgical exposure of impacted canines. The clinical outcomes evaluated in each patient were post-operative pain, bleeding during surgical procedure. In our study, clinical evaluation of outcomes in both categories revealed reduced pain at 1 hour and 24 hour after surgical procedures in patients who underwent surgery with LASER.. Soft tissue Laser can be an alternative to conventional surgery for soft tissue surgeries in orthodontics with better pain relief and reduced bleeding.

## Background:

A Light Amplified Stimulated Emission of Radiation (LASER) is a type of light wavelength that concentrates energy by traversing a collimated tube. Light is then used to convey this energy [1, 2- 3]. LASER radiation can be produced using a wide range of elements found in the periodic table. Certain LASER wavelengths-2,780 nm and 2,940 nm-are effective on soft tissues as well as hard tissues [4, 5-6]. On the contrary hand, other LASERs, like the 810 nm diode, are only effective on soft tissues while producing excellent surgical and hemostatic effects on them after soft tissue surgeries [7, 8-9]. In addition, the soft tissue diode LASERs function exceptionally well in incisions, with a tissue cutting depth that ranges from 2-4 mm [10, 11-12]. This gives them an edge over traditional surgery since the small blood and lymphatic veins are sealed, leading to hemostasis and less postoperatively oedema [13, 14-15]. As a consequence of regional heating, the formation of an eschar layer, and less scarring from post-operative tissue shrinking, the targeted tissues are also cleaned. As a result, sutures are no longer needed [16, 17, 18-18].

LASER therapy has been the subject of numerous researches as an auxiliary method in orthodontic practice [11- 14]. Numerous benefits of LASER therapy have impacted orthodontic procedures, including excision of soft tissue, detaching ceramic brackets, and expediting tooth movement. When it comes to soft tissue surgical procedures, a LASER is more advantageous than a scalpel. It removes post-operative sutures, disinfects the target area, and precipitates blood vessels [14-16].Studies have shown a decrease in analgesic along with local anesthetic drug consumption during LASER surgery, as well as a decrease in post-operative discomfort and soreness. Nevertheless, there are still several drawbacks to orthodontic adjunctive treatment using LASERs [17- 19]. The costly expense of LASER devices limits the application of LASERs. With regard to its tactile sensation, a scalpel is also preferred by some clinicians [20, 21- 22]. An additional problem with inexperienced operators is soft tissue ablation. Insufficient operational knowledge could expose bones and cause significant thermal injury to the tissue. According to several studies, there is no distinction between LASER and traditional scalpel procedures [23, 24- 25]. Erbium as well as diode LASERs are common varieties of LASER used in soft tissue treatments. Every LASER has a range of wavelengths along with input powers that allow it to be utilized for both hard and soft tissue surgery [3-7]. Because diode LASERs have an elevated absorption in soft tissue and minimal absorption in bone and other hard tissues, they are nearly usually employed for soft tissue ablation. Because of this characteristic, diode LASER degradation to hard tissue is reduced [7-10]. An orthodontic appliance in the mouth prevents good oral hygiene, which leads to plaque buildup and the potential for periodontal tissue inflammation [23, 25, 26- 27]. Gingival hypertrophy may ensue, particularly in patients with inadequate dental hygiene, necessitating aesthetic reconstruction or gingivectomy for correction [12-16]. Different types of soft tissue surgery are needed in orthodontics for other operations such as surgical exposure of impacted teeth, maxillary frenectomy, and operculectomy [7-11]. Numerous studies have looked into the numerous applications of LASER-assisted soft tissue ablation that are connected to orthodontic therapy in the past few years [10-15]. The majority of studies are case reports or case series with a small number of participants, making it impossible to compare LASER with more conventional methods [6-10]. Therefore, it is of interest to assess the clinical evaluation of LASER assisted soft tissue procedures for orthodontic treatment.

## Materials and Methods:

47 Orthodontic patients requiring soft tissue surgical procedures were split into two distinct categories based on the sort of therapy they had, and stratified random sampling was utilized for them. Category 2 underwent traditional surgery, while category 1 received treatment with a soft tissue 810 nm diode LASER (Table 1). In the orthodontic facility single orthodontist (INI) carried out the LASER procedures, and in the oral surgery facility the single surgeon carried out the surgeries that were performed.

## Requirements for inclusion:

[1] People in good health undergoing orthodontic treatment with a fixed appliance

[2] Orthodontic patients whose fixed appliance therapy has resulted in overgrown gingivae

[3] Patients undergoing orthodontics who have malocclusion because to improper frenal attachment

[4] Patients in orthodontics who need to be exposed surgically due to impacted teeth

## The following illnesses precluded participants from the study:

[1] Patients not receiving orthodontics treatment

[2] Orthodontic clients that don't practice good dental hygiene

[3] Orthodontic individuals infected in the mucogingival tract

[4] Orthodontic patients who have restricted mouth opening and trismus

[5] Patients in orthodontics who have any illness that interferes with the healing of wounds

The soft tissue surgical procedures carried out were aesthetic recontouring, gingivectomy, maxillary frenectomy, operculectomy and surgical exposure of impacted canines. Patients in the LASER group received treatment using an 810-nm diode LASER with a 400-µm fiber at 0.9-W power for 30 seconds per tooth. In the LASER group, the region was topically applied with TAC 20 gel (20% lidocaine, 4% articaine, 2% phenylephrine) to achieve local anesthetic. Patients would be questioned regarding any pain or discomfort they felt both during and right after the procedure. Until the operation was carried out under total local anesthesia, patients would be given an infiltration injection at their request, consisting of 2% lidocaine + 1:100000 epinephrine, if they felt any pain. No patient in the LASER group requested further anesthetic. Similar to the LASER group, topical TAC 20 gel was administered to the traditional surgery group. It was insufficient, and the patients complained of discomfort. They were given a 2% lidocaine infiltration injection (1:100000 adrenaline). In order to ensure that the procedure was performed under complete anesthesia, the surgeon continually inquired about the patient's perception of pain or discomfort.

The clinical outcomes evaluated in each patient were post-operative pain, bleeding during surgical procedure and after surgical procedure, need of additional anesthetic infiltration, need of suturing, need for scalpel incision in between procedures and analgesics consumption. The WHO bleeding scale was used to measure intraoperative and postoperative hemorrhage.

Grade 0: no bleeding

Grade one: bleeding in form of petechiae;

Grade two: minimal hemorrhage (clinically noteworthy);

Grade three: Gross blood loss requiring transfusion;

Grade four: Catastrophic blood loss linked to death, either retinal or cerebral.

Pain was measured using the Visual Analogue Scale (VAS) on a scale from 0-5. VAS 1 was recorded at 1 hour of procedure and VAS 2 after 24 hour from procedure.

## Statistical analysis:

Version 21.0 of the statistical software for social sciences (SPSS) was used to conduct the statistical analysis. To determine significance at a 95% confidence level, the test known as the chi-squared test was utilized. P-values, or probability values, of 0.05 or less were considered significant.

## Results:

22 patients (46.81%) patients underwent soft tissue surgeries with conventional surgical methods while 25 patients ((53.20%) patients underwent surgery with soft tissue 810 nm diode LASER. The soft tissue surgeries conducted by both procedures were aesthetic recontouring, gingivectomy, maxillary frenectomy, operculectomy and surgical exposure of impacted canines (Table 2). Clinical evaluation of outcomes in both categories revealed reduced pain at 1 hour and 24 hour after surgical procedures in patients who underwent surgery with LASER. Assessment of intraoperative bleeding revealed decreased bleeding in LASER assisted as compared to conventional surgery. Similarly post-operative bleeding was also low in LASER group. It was also observed that need for additional local anesthetic infiltration was greater n conventional surgery group. The requirement of placing suture after surgery was found low in case of LASER treated patents. While carrying out LASER assisted surgeries, need for incision between the procedures was quite low as compared to conventional surgery. The frequency of patients taking analgesics after surgical procedures was lesser in patients treated with soft tissue Diode LASER. The findings were significant statistically (Table 3).

## Discussion:

Orthodontic appliance obstructs proper dental hygiene, increasing the risk of periodontal tissue irritation and plaque accumulation [20-27]. Gingival hypertrophy may develop, especially in people with poor oral hygiene, and this can be corrected via gingivectomy or aesthetic restoration. Various forms of soft tissue surgery are required in orthodontics for additional procedures such operculectomy, maxillary frenectomy, and surgical exposure of impacted teeth [10-14]. Reports have examined the many uses of LASER-assisted soft tissue ablation related to orthodontic therapy [11-16]. It is tough to compare LASER with more traditional treatments because most research is case reports or case series with limited numbers of participants [5-10]. This study was therefore conducted to assess the clinical evaluation of LASER assisted soft tissue procedures for orthodontic treatment. In our study, clinical evaluation of outcomes in both categories revealed reduced pain at 1 hour and 24 hour after surgical procedures in patients who underwent surgery with LASER. Intraoperative bleeding had decreased bleeding score (0.31) in LASER as compared to conventional surgery (1.14). Similarly post-operative bleeding was also low in LASER group.t was also observed that need for additional local anesthetic infiltration was greater n conventional surgery group.

The findings of this study are supported by findings of other studies that revealed reduced discomfort, pain, intraoperative bleeding on applying LASER for soft tissue surgeries [12-19]. Many studies have looked into LASER therapy as an adjunctive treatment in orthodontics [11- 17]. LASER therapy has influenced orthodontic procedures in several ways, including as removing soft tissue, releasing ceramic brackets, and accelerating tooth movement. A LASER is a better tool than a knife for soft tissue surgical procedures [4-9]. It precipitates blood vessels, cleans the targeted area, and removes sutures left behind from surgery. Research has indicated a reduction in the need of analgesic and local anesthetic drugs during LASER surgery, in addition to a decrease in discomfort and soreness following the procedure [3- 7]. The findings of our study are not similar to some studies [19- 27]. Numerous investigations have found no difference between standard scalpel treatments and LASER procedures. However, there are still a number of disadvantages to LASER-assisted orthodontic adjunctive treatment. The use of LASERs is restricted by the high cost of LASER equipment [12-17]. Some professionals also prefer a scalpel because of its haptic sensation. Soft tissue ablation is another difficulty that arises with novice operators. Inadequate operational understanding may result in bones being exposed and severe heat damage to the tissue [14-21]. Common LASER types utilized in soft tissue treatments are diode and erbium LASERs. Every LASER can be used for both soft tissue and hard tissue surgery because of its spectrum of wavelengths and input powers [12-17]. Diode LASERs are almost exclusively used for soft tissue ablation because to their high absorption in soft tissue and low absorption in bone and other hard tissues. This property reduces the degradation of diode LASERs to hard tissue [13- 20]. It was also observed in our study that need for additional local anesthetic infiltration was greater n conventional surgery group. The requirement of placing suture after surgery was found low n case of LASER treated patents. While carrying out LASER assisted surgeries, need for incision between the procedures was quite low as compared to conventional surgery. The frequency of patients taking analgesics after surgical procedures was lesser in patients treated with soft tissue Diode LASER. The findings were significant statistically. The findings are in accordance with findings of other studies conducted involving soft tissue LASER [11-19]. A particular wavelength of light that concentrates energy by passing through a collimated tube is called a LASER. Then, this energy is communicated through light. Many elements from the periodic table can be used to create LASER radiation [21-27]. There are two LASER wavelengths that work well on both hard and soft tissues: 2,780 nm and 2,940 nm. However, some LASERs, such as the 810 nm diode, are limited to working on soft tissues, even if they have excellent hemo-static and surgical effects on them during soft tissue procedures [2-9]. Furthermore, the diode LASERs for soft tissue work incredibly well in incisions, severing tissue as deep as 2-4 mm [4- 10].

Due to the sealing of the small blood and lymphatic veins, which results in hemostasis and less postoperative oedema, they have an advantage over standard surgery [12-19]. The targeted tissues are additionally cleansed as a result of localized heating, the development of an eschar layer, and decreased scarring from post-operative tissue shrinkage. Sutures are therefore no longer required [4-11]. Many studies have looked into LASER therapy as an adjunctive treatment in orthodontics [19- 27]. Several advantages of LASER therapy have affected orthodontic processes, such as the removal of soft tissue, the removal of ceramic brackets, and the acceleration of tooth movement [10- 16]. A LASER is a better tool than a knife for soft tissue surgical procedures. It precipitates blood vessels, cleans the targeted area, and removes sutures left behind from surgery [5- 12].

## Conclusion:

Soft tissue LASER can be an alternative to conventional surgery for soft tissue surgeries in orthodontics with better pain relief and reduced bleeding.

## Figures and Tables

**Table 1:** Distribution of study participants

**Category**	**Intervention**	**Number of participants**
Category 1	Surgery with soft tissue 810 nm diode LASER	25
Category 2	Conventional surgery	22

**Table 2:** Details of Soft tissue procedures in orthodontic patients carried out by LASER and conventional surgery

	**Aesthetic recontouring No (%)**	**Gingivectomy No (%)**	**Maxillary Frenectomy No (%)**	**Operculectomy No (%)**	**Surgical exposure No (%)**	**Total**
Conventional surgery	2 (9.09)	4 (1.19)	4 (1.19)	2 (9.09)	10 (45.45)	22 (46.81)
LASER surgery	3 (12)	4 (16)	4 (16)	2 (8)	12 (48)	25 (53.20)
Total	5 (10.64)	8 (17.02)	8 (17.02)	4 (8.51)	22 (46.81)	47

**Table 3:** Comparison of outcomes between LASER surgery and conventional surgery in soft tissue surgical procedure in orthodontics patients

	**LASERs**	**Conventional Surgery**	**P value**
VAS (1hr after procedure) (Mean±SD)	2.4±0.34	4.6±0.21	0.001
VAS 2 (24 hr after procedure) (Mean±SD)	0.3± 0.04	2.4±0.17	0.001
Intra operative Bleeding	0.31	1.14	0.001
Post operative bleeding	0.01	0.97	0.001
Need of additional anesthetic infiltration	6.01%	96.23%	0.001
Need of suturing	0.01%	98.14%	0.001
Need for scalpel incison in between procedures	0.31%	67.21%	0.001
Analgesics consumption	1.49%	97.13%	0.001
